# Prediction of lymph node metastasis in T1 colorectal cancer based on combination of body composition and vascular invasion

**DOI:** 10.1007/s00384-024-04653-4

**Published:** 2024-06-03

**Authors:** Shizhen Zhou, Qinggang Yuan, Lixiang Liu, Kai Wang, Ji Miao, Hao Wang, Chao Ding, Wenxian Guan

**Affiliations:** 1 Department of General Surgery, Nanjing Drum Tower Hospital, Affiliated Hospital of Medical School, Nanjing University, Nanjing, 210008 China; 2 Department of General Surgery, Nanjing Drum Tower Hospital Clinical College of Xuzhou Medical University, Nanjing, 210008 Jiangsu China; 3 Department of General Surgery, Nanjing Drum Tower Hospital Clinical College of Nanjing Medical University, Nanjing, 210008 Jiangsu China; 4 Department of General Surgery, Nanjing Drum Tower Hospital Clinical College of Nanjing University of Chinese Medicine, Nanjing, 210008 Jiangsu China

**Keywords:** Colorectal cancer, CT, Skeletal muscle, Visceral fat, Vascular invasion

## Abstract

**Objectives:**

Lymph node metastasis (LNM) in colorectal cancer (CRC) patients is not only associated with the tumor’s local pathological characteristics but also with systemic factors. This study aims to assess the feasibility of using body composition and pathological features to predict LNM in early stage colorectal cancer (eCRC) patients.

**Methods:**

A total of 192 patients with T1 CRC who underwent CT scans and surgical resection were retrospectively included in the study. The cross-sectional areas of skeletal muscle, subcutaneous fat, and visceral fat at the L3 vertebral body level in CT scans were measured using Image J software. Logistic regression analysis were conducted to identify the risk factors for LNM. The predictive accuracy and discriminative ability of the indicators were evaluated using receiver operating characteristic (ROC) curves. Delong test was applied to compare area under different ROC curves.

**Results:**

LNM was observed in 32 out of 192 (16.7%) patients with eCRC. Multivariate analysis revealed that the ratio of skeletal muscle area to visceral fat area (SMA/VFA) (OR = 0.021, *p* = 0.007) and pathological indicators of vascular invasion (OR = 4.074, *p* = 0.020) were independent risk factors for LNM in eCRC patients. The AUROC for SMA/VFA was determined to be 0.740 (*p* < 0.001), while for vascular invasion, it was 0.641 (*p* = 0.012). Integrating both factors into a proposed predictive model resulted in an AUROC of 0.789 (*p* < 0.001), indicating a substantial improvement in predictive performance compared to relying on a single pathological indicator.

**Conclusion:**

The combination of the SMA/VFA ratio and vascular invasion provides better prediction of LNM in eCRC.

**Supplementary Information:**

The online version contains supplementary material available at 10.1007/s00384-024-04653-4.

## Introduction

Colorectal cancer is a prevalent gastrointestinal malignancy with an annual global incidence of approximately 1.8 million individuals. The implementation of a population-based screening programs for CRC has led to an increased detection rate of early stage (T1) CRC (eCRC) [1–3]. The treatment and prognosis of eCRC primarily depend on the presence or absence of lymph node metastasis (LNM) at the time of diagnosis [4, 5]. Most eCRC can be effectively treated with endoscopic submucosal dissection (ESD) [6, 7]. However, in cases where the pathological evaluation reveals high-risk indicators for LNM, such as submucosal infiltration depth (≥ 1000 μm), vascular invasion, or poorly differentiated histology, additional surgical treatment may be necessary [8–10].

According to research, only 8–16% of patients with eCRC who undergo surgical resection actually have LNM [11–14]. This suggests that approximately 80% of eCRC patients may potentially be subjected to unnecessary surgical intervention. Accurate predicting of LNM prior to ESD or surgical procedures is challenging. Consequently, it is a common practice among medical professionals to favor less invasive endoscopic interventions, even in situations where surgical intervention is actually necessary. However, this approach carries drawbacks for both patients and medical professionals. The precise preoperative detection of LNM in eCRC patients is important in prognostic assessment and guiding for reasonable therapeutic strategies. Therefore, it is imperative to identify an accurate marker for early prediction of LNM prior to treatment.

Currently, the focus of determining lymph node metastasis in eCRC lies primarily on the tumor itself, considering factors such as the depth of tumor infiltration and tumor differentiation. However, the significance of important nutritional and immune status, both at the whole body and local tumor level, is often overlooked. This oversight contributes to the limited predictive accuracy of existing methods. Previous research has established a correlation between elevated neutrophil-to-lymphocyte ratio (NLR) and other inflammatory marker abnormalities with sarcopenia, as well as their potential as prognostic indicators [15]. Furthermore, prior studies have illustrated the ability of inflamed adipose tissue to facilitate LNM in eCRC and serve as a marker for the overall immune status in CRC patients [15–20]. The objective of this study was to assess the association between skeletal muscle and adipose tissue status, as determined through clinical methods, and LNM in patients diagnosed with T1 CRC.

Contrast-enhanced computed tomography (CT) is suggested by the National Comprehensive Cancer Network guidelines as recommended preoperative evaluations for CRC [4]. However, the accuracy of using CT to identify positive lymph nodes for determining N staging is poor, with diagnostic accuracy ranging from 59 to 68% [21, 22]. Additionally, this method heavily depends on the subjective interpretation of doctors. With consideration of such limitations, we conducted a study to investigate the feasibility of using CT images to precisely assess lymph node status.

Consequently, the present study aimed to investigate the potential of CT measurement of skeletal muscle and adipose tissue areas for the accurate evaluation of lymph node status in eCRC.

## Methods

### Patients

**Fig. 1:**
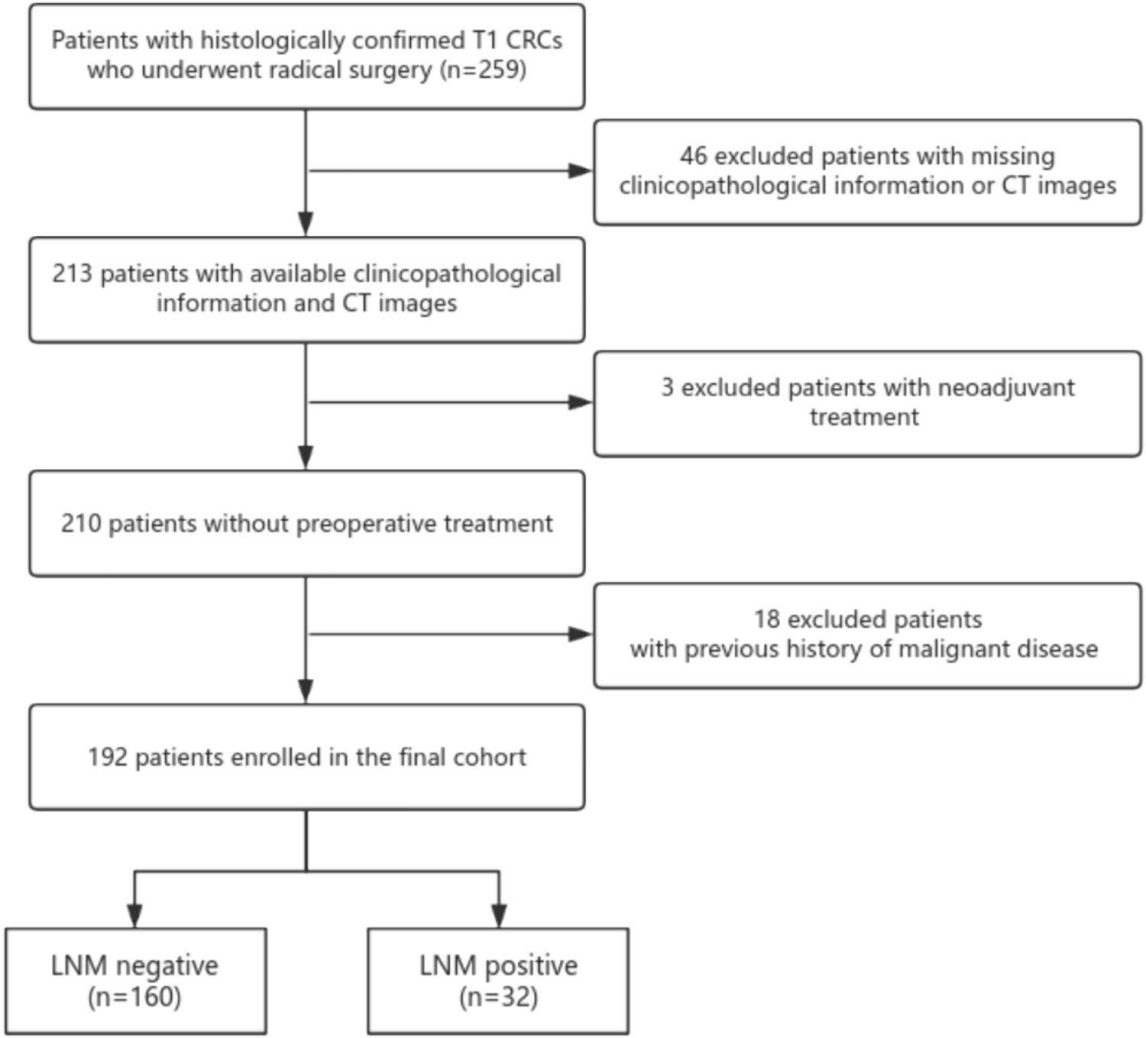
The screening flowchart

This single-center retrospective study received study-specific institutional review board approval. This study followed STROBE reporting guidelines (see S1 STROBE Checklist). Between June 2018 and March 2023, 259 consecutive eligible patients with primary colorectal cancer in Nanjing Drum Tower Hospital were selected. The inclusion criteria were as follows: [a] confirmed by histopathology as T1 stage colorectal adenocarcinoma; [b] underwent curative surgery (R0) and lymph node dissection; and [c] underwent a CT scan carried out less than 2 weeks before the surgery. The exclusion criteria were as follows: [a] imaging examination shows obvious enlarged lymph nodes; [b] pathological tumor stage Tis, T2, T3, or T4; [c] distant metastasis; [d] two or more primary tumors; [e] accepted preoperative treatment; and [f] a previous history of malignant disease. A total of 192 patients were included in the study (Fig. 1). The patients were categorized into two groups: LNM-positive and LNM-negative groups, based on the presence or absence of pathological confirmed LNM.

### Clinical and pathological characteristics

Retrieve clinical information such as gender, age, body mass index (BMI), carcinoembryonic antigen (CEA), CA199, white blood cell (WBC), glucose (GLU), urea nitrogen, creatinine, cholesterol, apolipoprotein A, apolipoprotein B and C-reactive protein(CRP) from electronic medical record systems. The postoperative pathological results, including tumor site, histological grade, depth of tumor infiltration, degree of vascular invasion, and tumor budding, were all reviewed by senior pathologists. All specimens were staged according to the 8th edition of the American Joint Commission on Cancer (AJCC) TNM staging system.

### Image acquisition and analysis

**Fig. 2:**
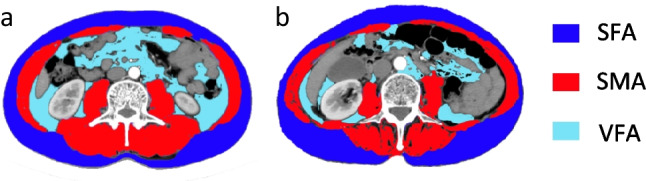
Measurement of the cross-sectional areas of skeletal muscle and fat at the L3 vertebral body level in CT scans of CRC patients with (**a**) and without (**b**) LNM. An axial CT image segmented into the visceral fat area (VFA), subcutaneous fat area (SFA), and skeletal muscle area (SMA)

All patients underwent abdominal CT examination in our institution within 2 weeks before surgery. Image J (version 1.53t) software was adopted for the measurement of the cross-sectional areas of skeletal muscle, subcutaneous fat, and visceral fat at the L3 vertebral body level in CT scans. An illustration is shown in Fig. 2, where the dark blue segment represents muscle tissue, the purple segment represents subcutaneous adipose tissue, and the light blue segment represents visceral adipose tissue. Additionally, the ratio of skeletal muscle to visceral fat area (SMA/VFA) was also calculated.

### Statistical analysis

Statistical analysis was conducted using SPSS (version 26.0). Continuous variables were presented as mean ± standard deviation (SD). Independent sample *t*-tests or Mann–Whitney *U*-tests were used to test the differences between continuous variables. Categorical variables were analyzed using Pearson’s chi-square (*χ*^2^) test, with appropriate continuity correction. Significant variables with a significance level (*p* < 0.1) in the univariate analysis related to LNM were further adjusted in the multivariate analysis using a logistic regression model. The multivariate logistic regression analysis was conducted to screen the independent risk factors for LNM. Utilizing logistic regression analysis to amalgamate various indicators into a novel predictive measure, considering the resultant value as a combined indicator. We used R packages “caret” and “stats” to perform *k*-fold cross-validation with *k* = 5 folds to evaluate the model performance. The diagnostic accuracy of LNM was evaluated by calculating the area under the receiver operating characteristics curve (AUROC) based on the selected factors. To compare the performance of different ROC curves, the Delong test was used. *p* values < 0.05 (two-sided) were considered statistical significance.

## Results

### Patients’ baseline characteristics

A total of 192 eligible patients were recruited for our study, with the majority being elderly individuals, having an average age of 60.4 years. More than half of the patients (54.8%) were male. The patients were divided into two groups based on LNM positivity, with a LNM positivity rate of 16.7%. There were no significant differences observed between the LNM-positive and LNM-negative groups in terms of age, gender, CEA, CA199, WBC, ALB, GLU, CRP, etc. (Table 1).

**Table 1:** Clinicopathologic characteristics

	LNM negative (*n* = 160)	LNM positive (*n* = 32)	*p* value
Gender			0.518
Male	85 (53.1%)	15 (46.9%)	
Female	75 (46.9%)	17 (53.1%)	
Age	60.3 ± 12.5	57.7 ± 11.0	0.259
BMI	24.2 ± 3.8	24.5 ± 3.9	0.748
WBC	5.9 ± 1.3	5.7 ± 2.1	0.900
ALB	41.3 ± 5	40.5 ± 3.0	0.434
GLU	4.9 ± 1	5.2 ± 1.2	0.212
CRP	5.7 ± 8.1	4.4 ± 5.3	0.373
Urea nitrogen	5.2 ± 1.4	4.7 ± 0.9	0.031
Creatinine	65.0 ± 15	58.1 ± 13.6	0.017
Cholesterol	4.4 ± 0.9	4.3 ± 0.9	0.886
Apolipoprotein A	1.0 ± 0.2	1.1 ± 0.3	0.373
Apolipoprotein B	0.7 ± 0.2	0.8 ± 0.2	0.560
CEA	1.6 ± 1.9	1.4 ± 1.5	0.648
CA19-9	12.3 ± 21.7	10.7 ± 6.1	0.683
Tumor size	24.3 ± 14.8	27.5 ± 26.1	0.347
Location of tumor			0.651
Colon	78 (48.8%)	17 (53.1%)	
Rectum	82 (51.2%)	15 (46.9%)	
Gross type of tumor			0.729
Non-ulcer	134 (83.8%)	26 (81.3%)	
Ulcer	26 (16.3%)	6 (18.8%)	
Differentiation			0.029
High	44 (27.5%)	10 (31.3%)	
Middle	104 (65.0%)	15 (46.98%)	
Low	12 (7.5%)	7 (21.9%)	
Vascular invasion			< 0.001
No	145 (90.6%)	20 (62.5%)	
Yes	15 (9.4%)	12 (37.5%)	
Tumor budding			0.066
0	64 (40%)	9 (28.1%)	
1	71 (44.4%)	12 (37.5%)	
2	17 (10.6%)	6 (18.8%)	
3	8 (5%)	5 (15.6%)	

*BMI* body mass index, *CEA* carcinoembryonic antigen, *CA199* carbohydrate antigen199, *WBC* white blood cell, *ALB* albumin, *GLU* glucose, *CRP* C-reactive protein

We conducted an analysis of postoperative pathological conditions in two patient groups and observed no statistically significant differences in tumor location, tumor type, tumor size, and tumor budding between the groups. However, we did find that the proportion of grade 3 tumor budding in the LNM-positive group was higher than that in the negative group. This difference, though not statistically significant, may be attributed to the small sample size. Tumor cell differentiation serves as an indicator of tumor malignancy, with poorly differentiated cells being more invasive. Our study revealed a significantly higher proportion of poorly differentiated patients in the LNM-positive group compared to the negative group. Vascular invasion has long been recognized as a risk factor for LNM, and our study further confirmed a higher incidence of vascular invasion in the LNM group.

### Muscle and fat characteristics

To examine the impact of skeletal muscle and fat on cancer cell behavior, we initially assessed the differences in blood indicators reflecting skeletal muscle and fat metabolism between the LNM-positive and LNM-negative groups (Table 2). The blood levels of creatinine and urea nitrogen, which indirectly indicate skeletal muscle levels, were significantly lower in the LNM-positive group. Interestingly, we did not observe any significant differences in blood indicators related to fat metabolism, such as cholesterol, apolipoprotein A, and apolipoprotein B, between the two groups.

**Table 2:** Skeletal muscle and fat area on L3 section of CT

	LNM negative (*n* = 160)	LNM positive (*n* = 32)	*p* value
SMA	134.1 ± 30.4	122.7 ± 30.5	0.055
SFA	133.4 ± 40.3	134.5 ± 24.3	0.882
VFA	88.1 ± 48.1	110.1 ± 31.4	0.014
SMA/VFA	2.0 ± 1.3	1.2 ± 0.4	< 0.001
SMA/SFA	1.1 ± 0.3	1.0 ± 0.4	0.071

*SMA* skeletal muscle area, *SFA* subcutaneous fat area, *VFA* visceral fat area, *SMI* skeletal muscle index

**Fig. 3:**
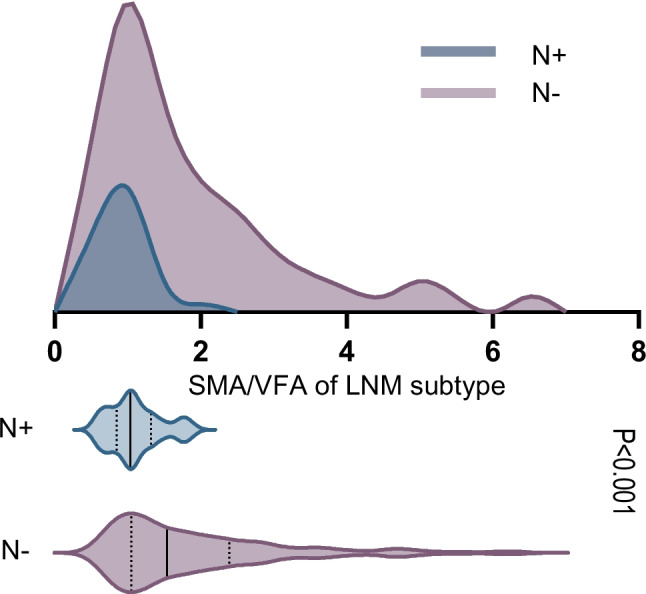
Distribution of SMA/VFA in LNM-positive and LNM-negative groups

We assessed the skeletal muscle and fat status of patients by directly measuring the skeletal muscle and fat area at the L3 level using CT images. We were able to differentiate between visceral fat and subcutaneous fat. Our findings revealed that the SMA in the LNM-positive group showed a decrease, although it was not statistically significant (134.1 ± 30.4 vs. 122.7 ± 30.5, *p* = 0.055). Interestingly, the content of VFA significantly increased in the LNM-positive group (88.1 ± 48.1 vs. 110.1 ± 31.4, *p* = 0.014), while there was no significant difference in SFA (133.4 ± 40.3 vs. 134.5 ± 24.3, *p* = 0.882). Combining the SMA with the VFA, we discovered that the ratio of these two indicators exhibited a more significant change between the LNM-positive and LNM-negative groups (2.0 ± 1.3 vs. 1.2 ± 0.4, *p* < 0.001) (Fig. 3).

### Multivariate analysis

Clinical factors were included in the logistic regression analysis, and those with a *p*-value less than 0.1 were included in the multivariate analysis. The results revealed that the SMA/VFA ratio (OR: 0.021, *p* = 0.007) and vascular invasion (OR: 4.074, *p* = 0.020) were identified as independent risk factors for lymph node metastasis. Tumor budding is commonly recognized as a risk factor for lymph node metastasis. Our multivariate analysis demonstrated that grade 3 tumor budding had an odds ratio of 6.373 (95% CI: 0.849–47.863), but the difference was not statistically significant (*p* = 0.072) (Table 3).

**Table 3:** Multivariate analyses of factors associated with lymphatic metastasis in patients with colorectal cancer

Variable	Multivariate analysis		
	OR (95%CI)		*p* value
SMA	1.009	0.975–1.044	0.612
VFA	0.974	0.942–1.006	0.111
SMA/VFA	0.021	0.001–0.352	0.007
Urea nitrogen	0.758	0.494–1.163	0.204
Creatinine	0.969	0.929–1.009	0.128
Vascular invasion			
No			
Yes	4.074	1.251–13.269	0.020
Tumor budding			
0			
1	1.468	0.468–4.607	0.510
2	2.889	0.623–13.387	0.175
3	6.373	0.849–47.863	0.072
Differentiation			
High			
Middle	0.632	0.214–1.868	0.406
Low	1.992	0.406–9.777	0.396

### Comparison of the predictive accuracy between independent prognostic factor

**Fig. 4:**
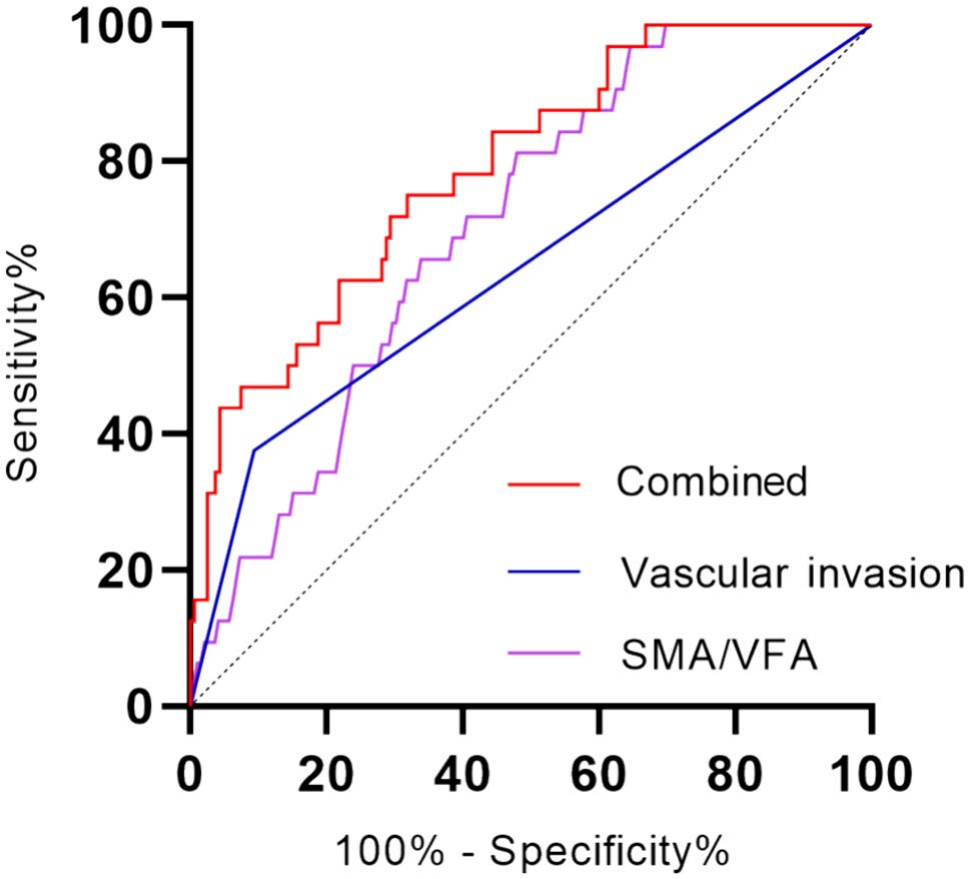
The receiver operating characteristic curves for predicting lymphatic metastasis

The classic pathological indicator of vascular invasion was first calculated, resulting in an AUROC of 0.641 (95%: 0.524–0.757, *p* = 0.012), indicating weak predictive power (Table 4, Fig. 4). Additionally, it has been observed that some doctors tend to make more aggressive judgments, leading to an increased false-positive rate and unnecessary surgeries for patients. However, the AUROC of SMA/VFA is 0.74 (95%: 0.668–0.826, *p* < 0.001), demonstrating a slight improvement in its predictive performance compared to pathological indicators. To further enhance the predictive model, we used binary logistic regression to predict SMA/VFA and vascular invasion as a new indicator called “Combined,” and observed that this indicator has a higher AUROC for LNM. Furthermore, employing fivefold cross-validation as a method of internal data validation to assess overfitting in the logical model yielded accuracies of 0.92, 0.87, 0.87, 0.82, and 0.84. These findings suggest that the model effectively captures patterns and similarities within unseen data. Remarkably, the AUROC of this model was found to be 0.789 (95%: 0.706–0.871, *p* < 0.001), and when compared to a single pathological indicator through DeLong test, the ROC was significantly better (*p* < 0.001). Therefore, by evaluating the content of skeletal muscle and visceral fat through CT scans, we can improve the ability to predict the presence of LNM using pathological methods.

**Table 4:** The AUROC of different indicators to predict lymph node metastasis

	AUC	95%CI	*p* value
SMA/VFA	0.740	0.668–0.826	< 0.001
Vascular invasion	0.641	0.524–0.757	0.012
Combined	0.789	0.706–0.871	< 0.001

## Discussion

This study explored the value of CT for the preoperative prediction of LNM in early-stage CRC. We utilized CT to measure the area of L3 plane visceral fat and skeletal muscle in eCRC patients to infer the lymph node metastasis status. Our results show that within our relatively small series, LNM status can be predicted with a good performance.

There exists substantial evidence substantiating the correlation between visceral obesity and the incidence and advancement of diverse tumors, notably colorectal cancer [23–25]. However, the linkage between visceral obesity and lymph node metastasis has only recently garnered attention in scholarly investigations. The release of inflammatory factors, such as TNF-a, IL-6, and MCP1, by the visceral fat of obese individuals induces localized chronic inflammation, thereby establishing a microenvironment that fosters tumor progression [26–29]. In this environment, tumor proliferation activity is activated, exhibiting more invasiveness, and the chances of tumor metastasis to lymph nodes increase [29, 30]. Brockmoeller et al. analyzed H&E slices of primary CRC through deep learning to identify inflamed fat, which can predict the status of lymph node metastasis in eCRC [18]. Due to the limitation of obtaining pathological results only after surgery, our study aims to investigate the feasibility of utilizing CT scans to evaluate the visceral fat status of patients prior to surgery and its potential in predicting the occurrence of LNM. Through our comprehensive analysis of the CT L3 level, it has been observed that LNM-positive patients exhibit a significantly larger visceral fat area, averaging 110, in contrast to LNM-negative patients who possess a relatively smaller visceral fat area of 88. However there is no significant correlation between SFA and LNM status, suggesting that visceral fat promotes tumor metastasis in the local microenvironment. This finding also highlights the limitations of using BMI as an indicator.

Sarcopenia are frequently observed in CRC patients and was associated with poor prognosis [31–34]. Previous studies have identified a noteworthy correlation between SMI and the infiltration of CD8 + cells within CRC tumors. Patients with high SMI exhibit a greater abundance of CD8 + T cells infiltrating the tumor in comparison to those with low SMI [20, 35]. Richards et al. found a clear correlation between muscle reduction in CRC patients and systemic inflammatory response [36]. Based on these findings, we can postulate that the condition of skeletal muscles might serve as an indicator of an individual’s immune response against tumor growth, consequently influencing the occurrence of lymph node metastasis in eCRC patients. In our thorough investigation, we assessed the skeletal muscle status by analyzing the skeletal muscle area in the L3 slice of CT scans. Intriguingly, our results revealed that the skeletal muscle area was notably smaller in CRC cases with positive LNM compared to those displaying negative LNM.

In our study, we investigated the association between skeletal muscle and visceral fat in predicting lymph node metastasis (LNM) in early colorectal cancer (eCRC). Previous research has introduced the concept of sarcopenic obesity (SOB), which refers to a decrease in skeletal muscle mass and strength accompanied by an increase in body fat [37–39]. However, SOB does not distinguish between subcutaneous fat and visceral fat. Our findings demonstrate that subcutaneous fat content is not correlated with LNM in eCRC. Considering that patients with positive lymph nodes have a smaller skeletal muscle area and a larger visceral fat area, we utilized a ratio to establish a connection between the two factors. Our results indicate that this ratio has a better predictive effect on lymph node metastasis compared to considering skeletal muscle or visceral fat alone.

The limitations of our study include a single-center setting, a small sample size, and the use of CT evaluation methods that may not be as precise. This is mainly due to the relatively low number of patients with T1 stage colorectal cancer, particularly those who have undergone surgery. While MRI is generally considered more effective in assessing muscle and fat in patients, it is not commonly performed on eCRC patients. In future research, we aim to enhance the CT evaluation method for skeletal muscle and visceral fat and develop machine learning models to improve the accuracy of predictions.

## Conclusion

The integration of the SMA/VFA ratio alongside vascular invasion yields enhanced prognostication of LNM in eCRC. Should our findings be corroborated by further investigations, the inclusion of skeletal muscle area and visceral fat area measurements in routine imaging reports, in conjunction with pathological indicators, holds significant promise for the prediction of lymph node metastasis.

## Supplementary Information

Below is the link to the electronic supplementary material.

Supplementary file1 (DOC 68 KB)

## Data Availability

The data supporting the results of this study can be reasonably requested by the corresponding author Ding Chao.
